# Results in Assisted Peritoneal Dialysis: A Ten-Year Experience

**DOI:** 10.1155/2015/712539

**Published:** 2015-10-27

**Authors:** Sara Querido, Patrícia Quadros Branco, Elisabete Costa, Sara Pereira, Maria Augusta Gaspar, José Diogo Barata

**Affiliations:** ^1^Department of Nephrology Centro Hospitalar do Médio Tejo, Avenida Xanana Gusmão, Apartado 45, 2350-754 Torres Novas, Portugal; ^2^Department of Nephrology, Centro Hospitalar de Lisboa Ocidental, Carnaxide, Portugal

## Abstract

*Background/Aims*. Peritoneal dialysis is a successful renal replacement therapy (RRT) for old and dependent patients. We evaluated the clinical outcomes of an assisted peritoneal dialysis (aPD) program developed in a Portuguese center. *Methods*. Retrospective study based on 200 adult incident patients admitted during ten years to a PD program. We included all 17 patients who were under aPD and analysed various parameters, including complications with the technique, hospitalizations, and patient and technique survival. *Results*. The global peritonitis rate was lower in helped than in nonhelped patients: 0.4 versus 0.59 episodes/patient/year. The global hospitalization rate was higher in helped than in nonhelped patients: 0.67 versus 0.45 episodes/patient/year (*p* = NS). Technique survival in helped patients versus nonhelped patients was 92.3%, 92.3%, 83.1%, and 72.7% versus 91.9%, 81.7%, and 72.1%, and 68.3%, at 1, 2, 3, and 4 years, respectively (*p* = NS), and patient survival in helped patients versus nonhelped patients was 93.3%, 93.3%, 93.3%, and 74.7% versus 95.9% 93.7%, 89%, and 82% at 1, 2, 3, and 4 years, respectively (*p* = NS). *Conclusions*. aPD offers an opportune, reliable, and effective home care alternative for patients with no other RRT options.

## 1. Introduction

In the last two decades, most developed countries have seen a continuous growth in the number of patients with end-stage renal disease (ESRD) commencing renal replacement therapy (RRT). It is possible to identify the main factors which influence this growth: aging of the population due to a greater life expectancy; increase in the incidence of chronic kidney disease related to age; better care of patients with chronic diseases; patients with physical limitations who survive now for longer periods of time; and developments in industry and biotechnology [1, 2]. In Portugal in 2014, almost 60% of patients starting dialysis were over the age of 65 years and only 8,73% of all incident patients started PD. Despite this increase in the number of elderly and dependent patients who need RRT [3], a decline in the utilization of peritoneal dialysis (PD) has occurred in a number of countries since the mid-1990s [4]. This decline is particularly acute for the elderly population [5]. Nevertheless, elderly and dependent patients benefit, specially, from PD as it would avoid travelling to dialysis centers, reduce hemodynamic instability [6], diminish the risk of central venous catheter-associated bacteremia [5], improve blood pressure control [5, 7], and diminish the bacterial translocation and myocardial stunning [8]. Such a population cohort is susceptible to several physical barriers (decreased strength to lift PD bags, decreased manual dexterity, and decreased vision, mobility, and hearing) and cognitive barriers (language, noncompliance, dementia, and psychiatric conditions). Thus, providing home care assistance to support those patients on PD may help increasing the number of individuals that can be safely treated at home [9] as well as reducing hazards related to personal limitations. Assistance to PD patients involves the identification and training of an individual (other than the patient) to perform dialysis-related tasks, such as connecting the patient to the cycler, setting up the cycler, disconnecting the patient from the cycler, or performing continuous ambulatory peritoneal dialysis (CAPD) exchanges. Since 1997 [10], following the publication of the first successful French experience (when home care nurses treated elderly patients with assisted CAPD) assisted PD (aPD) became a valuable alternative to provide successful RRT for old and dependent patients. Over the past decade, 11,557 patients started PD in France, out of which 44.6% have been on aPD [11]. The nurses who assist at home in France are paid directly by patients, who are partially reimbursed by the French healthcare insurance. Elderly patients are also successfully treated with PD in Hong Kong, where PD is the first treatment option. In March 2007, 80% of patients (median age: 62.3 years) were on PD [12]. Presently aPD is a dialysis modality in evolution all over Western Europe, Canada, South America, and Asia [11–16].

Although aPD is a valuable and successful renal replacement therapy for old and dependent patients we have to consider and face the lack of social support for patients in our country. The aim of this study was to evaluate the results of the aPD program, offered as first option or last resort to elderly or physically incapable end-stage renal disease patients, considering the clinical outcomes of this technique in a single Portuguese center.

## 2. Material and Methods

This is a retrospective study performed at a single PD Unity in Portugal (PD Unity of Hospital de Santa Cruz, Carnaxide, Portugal), based on the study of 200 adult incident patients admitted during 10 years (2004–2014) to the PD program. We included and studied a total of 17 patients with physical or cognitive debilities who were under aPD. Assisted-care patients were defined as patients who are unable to perform peritoneal fluid exchange at the beginning of PD or who lack the ability to perform their own treatment and have, therefore, to rely on nonprofessional care, including family members or domestic workers. We analyzed demographic, clinical, and laboratory parameters, complications with the technique, hospitalizations, and patient and technique survival through research in clinical processes. The degree of dependence was analyzed through the application of the Davies Score [17] and Karnofsky Index. Normally distributed variables were expressed as mean ± standard deviation and nonnormally distributed variables were expressed as frequency and percentage. Unadjusted analysis was performed by the Kaplan-Meier method to analyse technique survival between self-care and aPD patients. Technique failure classified the dropout from aPD to Hemodialysis due to peritoneal membrane failure or peritonitis. The technique survival was defined in patients who remained on aPD during the observation period and kidney receptors allograft and patients who died during the aPD program due to any reason other than peritonitis or peritoneal membrane failure.

All statistical tests were performed using the Statistical Package for the Social Sciences (SPSS) 14.0 software (SPSS, Inc., Chicago, IL, USA). Categorical variables were described as numbers or percentage of relative frequencies and quantitative variables as mean ± standard deviation (SD) for continuous normally distributed variables. Cox regression was used to compare survival rates.

Differences between clinical data were assessed by Student's *t*-test for paired samples for normal variables and paired Wilcoxon test for continuous data with nonnormal distribution. A *p* value of < 0.05 was considered to be statistically significant.

## 3. Results

We followed a cohort of 17 consecutive incident patients who were engaged in aPD from January 2004 to October 2014. Median age was 58 ± 20 years; 9 patients were men, 12 patients had hypertension, and 6 had diabetes. Fourteen patients had only one helper, like a close relative; 2 patients were treated by multiple family members; and one patient received treatment from 2 home assistance employees. One patient was on PD due to vascular access failure; 5 patients chose PD; and for 11 patients PD was a family's choice. Five patients had physical and cognitive limitations ab initium. The mean age of the ones who had physical limitations was 35.2 years; patients with cognitive limitations had a mean age of 65 years; 7 patients were treated with automated assisted peritoneal dialysis (APD) and 10 patients with CAPD. The Davies Score was greater than 2 in 52.9%; Karnofsky Index was less than 70 in 64.7%. The patients were under PD for 36.98 ± 31.43 months; 4 of them had an acute onset of the technique; kt/V weekly was 2.22 ± 0.60 and nPCR was 0.88 ± 0.30 g/Kg/day. Peritoneal equilibration test (PET) was performed in 14 patients: 8 were low-average and 6 were high-average transporters. The residual renal function was 3.02 ± 3.85 mL/min/1.73 m^2^ and 3 patients were anuric. Demographic and PD related parameters in patients under autonomous PD and aPD are compared in Table 1.

**Figure 1:**
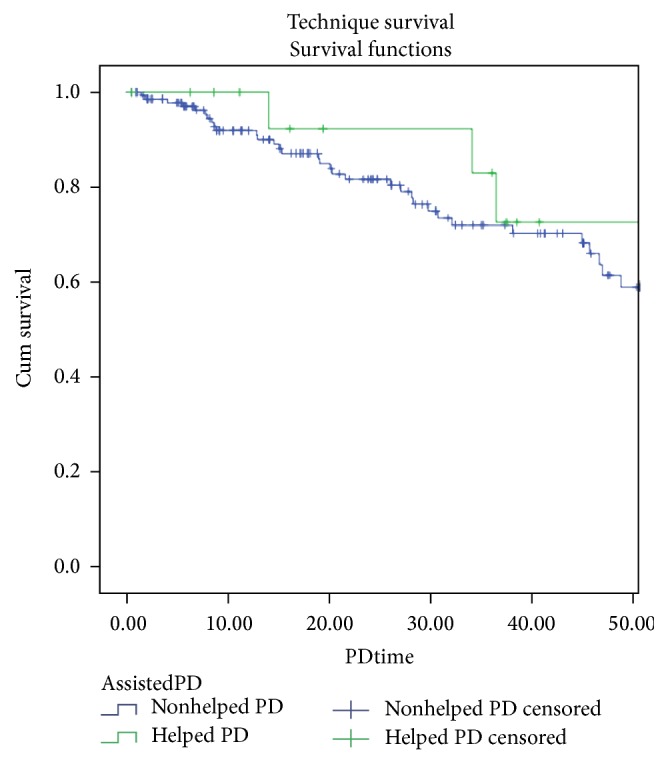
Technique survival in helped and nonhelped patients.

**Figure 2:**
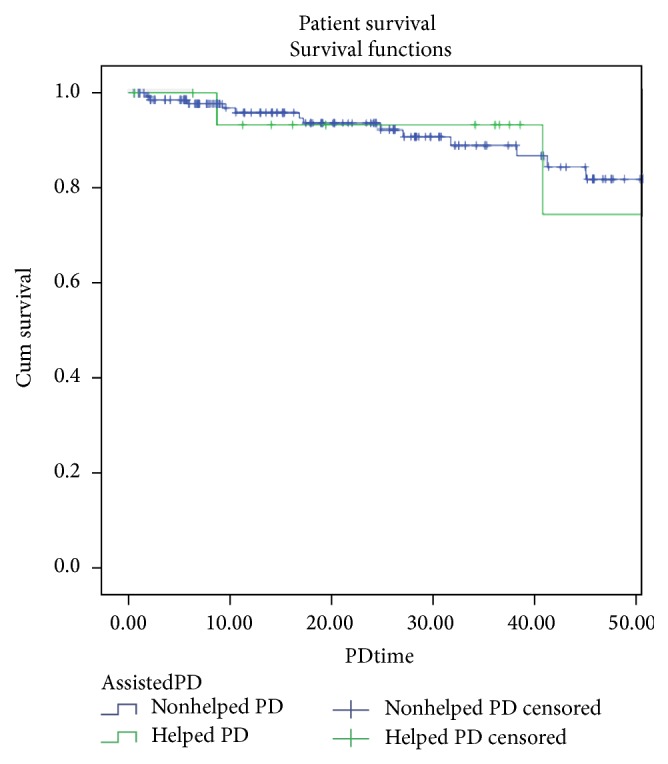
Patient survival in helped and nonhelped patients.

Half of the patients have never had a peritonitis episode; 2 patients had a tunnel infection; and 9 patients had one or more episodes of exit-site infection. Six patients needed more than 1 Tenckhoff catheter; 4 patients died during PD technique; 3 patients started haemodialysis (1 due to PD membrane failure and 2 due to peritonitis); and 1 patient received a kidney allograft. The global peritonitis rate was lower in helped than in nonhelped patients: 0.4 versus 0.59 episodes/patient/year. The global hospitalization rate was higher in helped than in nonhelped patients: 0.67 versus 0.45 episodes/patient/year (*p* = NS). Technique survival in helped patients versus nonhelped patients was 92,3%, 92.3%, 83,1%, and 72.7% versus 91,9%, 81,7%, 72,1%, and 68,3%, at 1, 2, 3, and 4 years, respectively (*p* = NS) (Figure 1) and patient survival in helped patients versus nonhelped patients was 93,3%, 93,3%, 93,3%, and 74,7% versus 95,9% 93,7%, 89%, and 82% at 1, 2, 3, and 4 years, respectively (*p* = NS) (Figure 2). Two patients remained on aPD for more than 7 years.

## 4. Discussion

The outcome of PD is usually assessed by patient survival, technique survival, and peritonitis incidence [18]. PD shows no difference in patient survival, technique survival, and peritonitis rate [19], between elderly and younger patients. Nonetheless, the outcome of assisted-care and self-care in elderly PD patients is not consistent. The RDPLF report showed that patients under assisted-care, either by family members or by nurses, had a poorer survival rate than self-care patients [9]. On the other hand, in a Hong Kong research, no significant differences were found in self-care elderly and nonelderly patients in terms of survival and technique survival. Our study showed that assisted-care PD patients had a poorer outcome in terms of patient survival (12th, 24th, and 48th months) and hospitalization rate but a better performance in terms of peritonitis incidence and technique survival. Lobbedez et al. [11] studied 36 aPD patients and observed a relatively high peritonitis rate, with 50% presenting at least 1 episode per year. Issad et al. showed that peritonitis and exit-site infection rates were not significantly different between aPD and self-care PD patients [10]. Verger et al. revealed that the probability of being peritonitis-free at 2 years was higher for patients assisted by a family member than for those assisted by a private nurse [14]. In our study, the global peritonitis rate was lower in helped than in nonhelped patients (0,4 versus 0,59 episodes/patient/year). As in Verger et al. study, this fact could be explained by the high dedication level of family members.

According to Lobbedez et al. [11] a higher percentage of aPD patients (79%) were hospitalized during the first follow-up year, with peritonitis being the most frequent cause of hospitalization. Our results are similar to the literature with a global hospitalization rate higher in helped than in nonhelped patients [0.67 versus 0.45 episodes/patient/year (*p* = NS)]. This fact could be explained by the high comorbidity index in the aPD patients.

There are not many studies concerning technique survival on assistance method [9, 18]. Those studies showed no association between technique survival and assistance method. We found that technique survival was better in aPD patients. It is difficult to interpret the facts due to the small number of patients in aPD program, but, globally, aPD did not show any disadvantage in terms of technique survival. Lobbedez et al. [11] reported 83% 1-year survival of aPD patients at their center. Povlsen et al. [16] showed that the 1-year and 2-year survival rates of functionally dependent elderly patients on aPD were 58% and 48%, respectively. In our study, the survival rate was 93,3% in the first 3 years under technique, with 2 patients remained on aPD for more than 7 years. The patient who died earlier was a dependent patient with a severe heart disease. Nevertheless, in our series, the survival rate in aPD patients is probably overestimated, considering that these patients had a median age of 58 years, much younger when compared with aPD patients from other series [10, 15].

In UK, aPD is in its infancy. There is no extra funding for providing assistance, so developing a service depends on local enthusiasm. The model of care being developed is based on aPD with one visit per day from a paid carer and the patient or family carrying out the connection and disconnection to/from machine. The community nursing service is not adequately staffed or funded to take on this extra role [20].

## 5. Conclusions

Our results compare favourably with international reports. In this clinical observation study, aPD offered an opportune, reliable, and effective home care alternative for patients with no other renal replacement therapy options. Due do the lack of support from social institutions the helpers were close relatives in almost every case. It is necessary to adopt measures and institutional support to care for these patients, not forgetting that some of them are young people with a considerable life expectancy, despite cognitive or motor deficits. Nevertheless, larger, longer, and better studies on aPD are warranted. Till now, studies have showed that assistance gives dependent patients an opportunity to have a home-based dialysis modality, increasing the number of patients who can choose an appropriate treatment despite their physical, cognitive, and social conditions.

## Figures and Tables

**Table 1:** Demographic and PD related parameters in patients under autonomous PD and aPD.

	Autonomous PD (*n* = 183)		Assisted PD (*n* = 17)	
Age (years)	55,7 ± 15,2		58 ± 20	

Male (*n*/%)	122 (66,67)		9 (52,94)	

Hypertension (*n*/%)	172 (93,99)		12 (70,59)	

Diabetes (*n*/%)	58 (31,69)		6 (35,29)	

APD (*n*/%)	64 (34,97)		7 (41,18)	

CAPD (*n*/%)	119 (65,03)		10 (58,82)	

Time under PD (months)	29,7 ± 22,7		36,98 ± 31,43	

Technique survival (months/%)	12 m	91,9	12 m	92,3
	24 m	81,7	24 m	92,3
	36 m	72,1	36 m	83,1
	48 m	68,3	48 m	72,7

Patient survival (months/%)	12 m	95,9	12 m	93,3
	24 m	93,7	24 m	93,3
	36 m	89	36 m	93,3
	48 m	82	48 m	74,7

Weekly kt/V	2,4 ± 0,70		2,22 ± 0,60	

nPCR (g/Kg/day)	0,93		0,88 ± 0,30	

PET	*N* = 175	D/P < 0,5 = 2%	*N* = 14	D/P 0,5–0,64 = 8
		D/P 0,5–0,64 = 35%		
		D/P 0,65–0,81 = 62%		D/P 0,65–0,81 = 6
		D/P > 0,81 = 1%		

Residual renal function (mL/min/1,73 m^2^)	*N* = 155	7,14 ± 11	*N* = 14	3,02 ± 3,85

APD: automated peritoneal dialysis; CAPD: continuous ambulatory peritoneal dialysis; PET: peritoneal equilibration test.
